# Psilocybin treatment extends cellular lifespan and improves survival of aged mice

**DOI:** 10.1038/s41514-025-00244-x

**Published:** 2025-07-08

**Authors:** Kosuke Kato, Jennifer M. Kleinhenz, Yoon-Joo Shin, Cristian Coarfa, Ali J. Zarrabi, Louise Hecker

**Affiliations:** 1 Division of Pulmonary, Allergy and Critical Care and Sleep Medicine, Department of Medicine, Emory University, Atlanta, GA USA; 2 Section of Pulmonary, Critical Care and Sleep Medicine, Department of Medicine, Baylor College of Medicine, Houston, TX USA; 3 Division of Cardiovascular Research, Department of Medicine, Baylor College of Medicine, Houston, TX USA; 4 Department of Molecular and Cellular Biology, Baylor College of Medicine, Houston, TX USA; 5 Division of Palliative Medicine, Department of Family and Preventive Medicine, Emory University School of Medicine, Atlanta, GA USA; 6 Atlanta VA Healthcare System, Atlanta, GA USA

**Keywords:** Senescence, Cell biology, Molecular biology

## Abstract

Psilocybin, the naturally occurring psychedelic compound produced by hallucinogenic mushrooms, has received attention due to considerable clinical evidence for its therapeutic potential to treat various psychiatric and neurodegenerative indications. However, the underlying molecular mechanisms remain enigmatic, and few studies have explored its systemic impacts. We provide the first experimental evidence that psilocin (the active metabolite of psilocybin) treatment extends cellular lifespan and psilocybin treatment promotes increased longevity in aged mice, suggesting that psilocybin may be a potent geroprotective agent.

## Introduction

To date, >150 clinical studies with psilocybin have been completed or are ongoing for various clinical indications, including psychiatric (anxiety, depression, addiction), neurodegenerative (Alzheimer’s), pain, and more^1–3^. Human studies have demonstrated that a single-dose of psilocybin can improve debilitating physical and psychological symptoms—with durable effects (up to ~5 years)^4,5^. Despite considerable clinical evidence supporting the therapeutic benefits of psilocybin, the molecular mechanisms responsible for these impacts remain enigmatic. Studies with psilocybin have predominantly focused on neurological impacts and/or behavioral outcomes; few studies have evaluated alternative or systemic mechanisms which may also contribute to its beneficial effects. The “psilocybin-telomere hypothesis”^6^ postulates that psilocybin interventions may quantifiably impact telomere length, which offers a potential explanation for its efficacy across a wide range of clinical indications. This hypothesis is based on a large corpus of studies linking mental health biological aging markers^6^. Accumulating evidence indicate that clinical depression accelerates aging and telomere shortening^7–9^. Positive mental psychological states are associated with longer telomeres, whereas negative psychological conditions (e.g. chronic stress, anxiety, and depression) are associated with telomere attrition^7,10–13^. Given the clinical evidence supporting the efficacy of psilocybin for these conditions, it is plausible that psilocybin may impact telomere length. However, no prior studies have experimentally investigated the direct impact of psilocybin on biological aging.

**Fig. 1:**
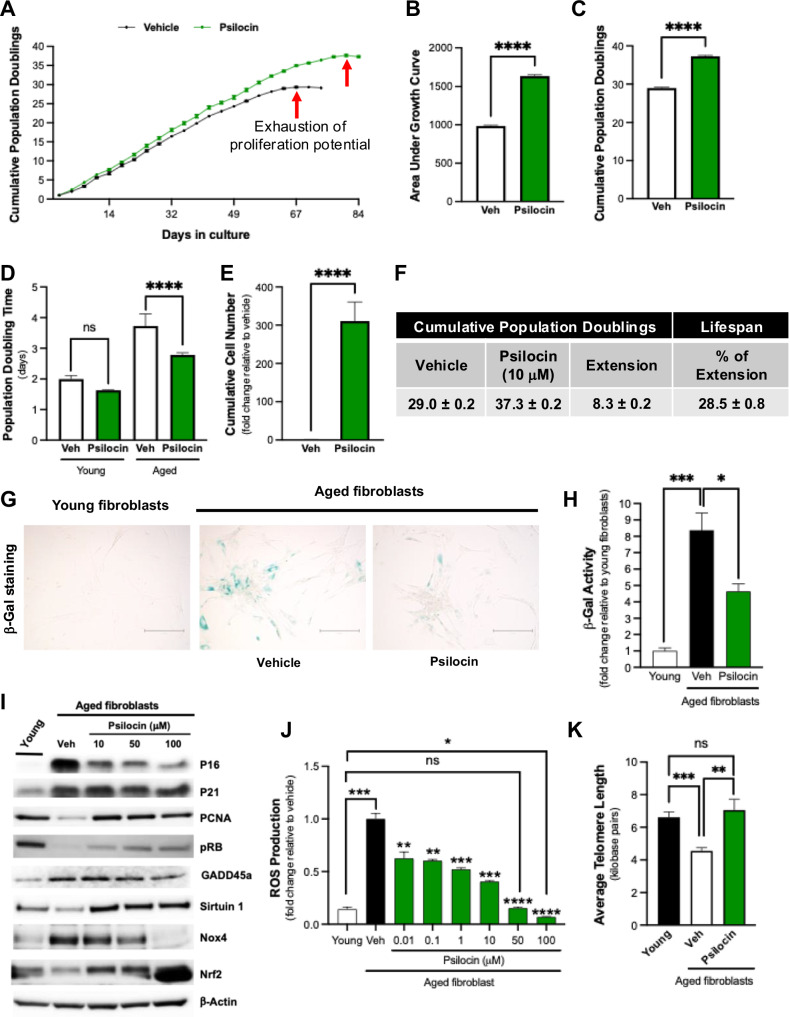
Psilocin treatment extends cellular lifespan. Human lung fibroblasts were treated continuously with vehicle (DMSO 0.02%) or 10 µM psilocin until they reached replicative senescence. **A** Cumulative population doubling curves; arrows indicate time point when cells reach replicative senescence (*n* = 4 technical replicates). **B** The area under the curve (AUC) was calculated using the sum of trapeze areas for each time point intervals (*n* = 4 technical replicates). **C** Cumulative population doublings at the onset of senescence (*n* = 4 technical replicates). **D** Population doubling time comparing young cells (0–4 days) vs. aged cells (60–63 days) post-treatment. Groups were compared using 2-way ANOVA (*n* = 4 technical replicates). **E** Cumulative cell number over cellular lifespan. **F** Table showcasing cellular lifespan population doublings and lifespan extension. Extension refers to the number of additional population doublings of psilocin-treated cells vs. vehicle, which is also shown as a percent increase relative to vehicle-treated cells. **G**, **H** Senescence was assessed at 42 days post-treatment by senescence-associated β-galactosidase (β-gal) staining; scale bar = 70 µm (**G**) and quantitative assessment of β-gal activity (*n* = 5 technical replicates) (**H**). **I** Western blot demonstrating dose-dependent alterations in protein expression of age-associated markers. **J** Reactive oxygen species (ROS) production was evaluated by Amplex Red assay (*n* = 3 technical replicates). **K** Average telomere length was assessed by RT-PCR (*n* = 4 technical replicates) and is expressed as kilobase pairs per chromosomal end. All values are shown as mean ± SD. Unless otherwise stated, comparisons were made using two-sided unpaired *t*-tests with unequal variance.; *p < 0.05, **p < 0.01, ***p < 0.001, ****p < 0.0001, ns not significant.

To evaluate the impact of psilocybin on cellular aging, we employed a validated model of replicative senescence using human fetal lung fibroblasts^14^. For all in vitro studies, we used psilocin (the active metabolite of psilocybin), which is formed when psilocybin is broken down after ingestion. Cells were serially passaged with media containing psilocin or vehicle until they reached replicative senescence. Psilocin treatment (10 μM) resulted in a 29% extension of cellular lifespan, characterized by delayed exhaustion of proliferative potential, increased cumulative population doublings, and decreased population doubling time, compared to vehicle (Fig. 1A–F). Results were more striking using a higher dose of psilocin in the same cell type (100 μM treatment led to a 57% extension in cellular lifespan; Supplementary Fig. 1A–F). Induction of senescence occurred in both vehicle and psilocin-treated cells, as both groups reached exhaustion of their proliferative potential (no evidence of oncogenic transformation was observed), however the onset of senescence was delayed in psilocin-treated cells (Fig. 1A). Further, compared to vehicle, psilocin-treated cells exhibited decreased βgal activity (Fig. 1G–H). These results were consistent with dose-dependent reductions in markers of cell cycle arrest (p21, p16), and increased markers of proliferation (PCNA) and DNA replication (pRB) (Fig. 1I). Compared to vehicle, psilocin treatment also led to elevated sirtuin1 (SIRT1; a critical role in regulating cellular aging, metabolism, and stress-responses) and decreased Growth Arrest and DNA Damage-inducible 45 alpha (GADD45a) levels, suggesting reduced DNA damage (Fig. 1I). Psilocin treatment also reduced oxidative stress levels in a dose-dependent manner (Fig. 1J), which was associated with decreased levels of NADPH oxidase-4 (Nox4, a master regulator of oxidant production) and increased nuclear factor erythroid 2-related factor 2 (Nrf2, a master regulator of antioxidant responses) (Fig. 1I). Overall, these results suggest that the in vitro impacts of psilocin are dose-dependent, with higher dosing ultimately leading to greater cellular life extension. To further validate these findings, we repeated these studies with a different cell type (adult human skin fibroblasts); 100 μM psilocin treatment increased cellular lifespan by 51%, which was accompanied by reduced senescence and decreased oxidative stress levels (Supplementary Fig. 2). To investigate other potential mechanisms by which psilocin contributes to increased lifespan, we also evaluated the impact on telomere length (reductions in telomere length is a hallmark of cellular aging). As expected, senescent vehicle-treated cells exhibited reduced telomere length compared to young control cells (Fig. 1K). In contrast, telomere length was preserved in psilocin-treated age-matched cells (Fig. 1K). In summary, these data suggest that psilocin impacts signaling pathways associated with cellular aging, which ultimately delayed the onset of senescence and increased cellular lifespan.

**Fig. 2:**
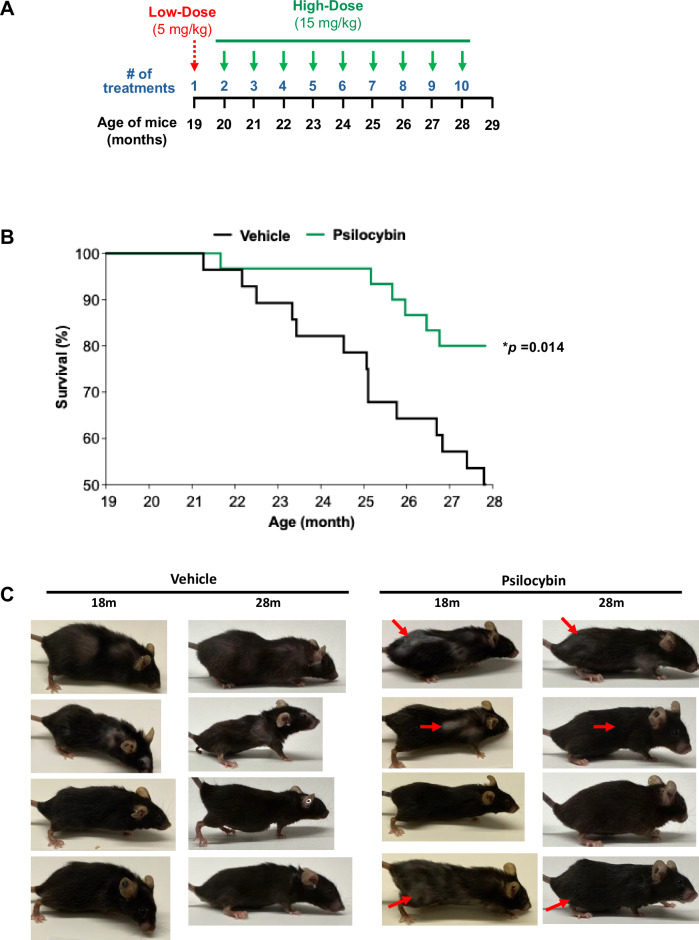
Psilocybin treatment in aged mice extends lifespan. C57BL/6J aged (19 month) female mice were treated with vehicle (*n* = 28) or psilocybin (*n* = 30) by oral gavage. Mice were given a lower dose (5 mg/kg) of psilocybin in month 1, followed by monthly dosing with high dose (15 mg/kg). At 10 months post-initial treatment, when the first group of mice reached median survival, all were euthanized. **A** Schematic diagram of treatment protocol. **B** Kaplan–Meier survival curve over the 10-month treatment duration, showing vehicle-treated (*n* = 14 out of 28) vs. psilocybin-treated (*n* = 24 out of 30) mice; *p = 0.014 using the Log-rank Mantel–Cox test. **C** Representative images of mice prior to treatment (19 month) and after the final treatment (28 month) with vehicle or psilocybin. Red arrows indicate regions where hair growth and/or hair color changes were observed.

To evaluate the impact of psilocybin on longevity in vivo, aged (19 month) female mice were treated with vehicle or psilocybin once/month for 10 months (Fig. 2A); mice were initially given a low-dose (5 mg/kg) for the first treatment followed monthly high-dose (15 mg/kg) treatment for a total of 10 treatments. We elected to utilize 19-month old mice, which is roughly equivalent to 60–65 human years, in order to evaluate its therapeutic potential as a clinically-relevant anti-aging intervention. Within 30 min post-treatment, mice exhibited increased head-twitch response (data not shown), which is a well-established behavioral indicator of hallucinogenic impacts of psilocybin in mice^15^. Both psilocybin and vehicle groups exhibited some loss in body weight from the start to end of the treatment protocol, however weight loss was not significantly different in vehicle- vs. psilocybin-treated mice (Supplementary Fig. 3). Notably, psilocybin treated mice demonstrated significantly higher survival (80%), compared to vehicle (50%) (Fig. 2B). Although not quantitatively measured, psilocybin-treated mice exhibited phenotypic improvements in overall fur quality, including hair growth and reductions in white hair compared to vehicle-treated mice (Fig. 2C). In summary, we provide the first experimental evidence demonstrating that psilocybin treatment can enhance survival in aged mice.

Psilocybin is a potent serotonergic agonist that interacts with the serotonin receptor (5-HT_2A_) and other 5-HT receptor subtypes. Notably, the 5-HT_2A_ receptor is expressed in multiple organs and cell types (including fibroblasts, neurons, cardiomyocytes, endothelial, epithelial, macrophages, and T-cells)^16^. A recent study demonstrated that 5-HT_2A_ stimulation in cortical neurons induced SIRT1-dependent expression of antioxidant enzymes, which led to reduced oxidative stress and neuroprotection^17^. SIRT1 is a key regulator of senescence, and overexpression of SIRT1 has been shown to extend organismal lifespan in C. elegans and mice^18^. Here, we demonstrate that psilocin increased SIRT1 expression in cells, suggesting a potential mechanism by which psilocybin delays senescence and promotes longevity. This study provides the first experimental evidence suggesting that psilocybin may impact multiple hallmarks of aging, including delayed senescence, preservation of telomere length, enhanced DNA stability (via increased DNA-damage responses such as GADD45a), and/or it could reduce aberrant intercellular communication (via decreased oxidative stress and subsequent signaling). Future studies are warranted to further elucidate the impacts of psilocybin on age-related pathways and the molecular mechanisms responsible for its systemic effects (including 5-HT-dependent and/or -independent mechanisms). Prior studies have demonstrated that the long-lasting impacts of psychedelic treatments may be due to epigenomic-driven alterations, including chromatin remodeling and DNA methylation^19–21^. It is possible that psilocybin may also mediate epigenetic changes which contribute to the observed geroprotective effects; such studies warrant investigation. However, regulatory barriers imposed by its schedule I designation along with the limited availability of federal funding for psilocybin research remain significant obstacles that have hindered research progress^22,23^; thus, the mechanisms underlying its potential therapeutic benefits remain poorly understood.

Our study provides the first experimental evidence demonstrating that psilocybin impacts hallmarks of aging, supporting the previously proposed “psilocybin-telomere hypothesis”^6^. We demonstrate that psilocin/psilocybin treatment extends both cellular and animal lifespan (even when treatment is initiated late in life). An effective anti-aging treatment that could be administered to adults during late life could have significant clinical potential. The dose utilized in mice was modeled based on a clinical trial in patients ranging from 29 and 70 years (three patients were >65 years), where no serious adverse events were reported at the study endpoint or the post-study follow-up (98 days)^24^; These findings support the feasibility of psilocybin treatment in older adults. Further, the FDA’s designation of psilocybin as a “breakthrough therapy” underscores its safety profile, as minimal adverse effects have been reported^25–28^. However, additional studies are warranted to identify optimized protocols for therapeutic efficacy, including the age of treatment initiation, frequency and dose of treatments, and to determine if treatment impacts maximal lifespan. Would earlier intervention yield greater therapeutic benefits, and/or is there a threshold in older age beyond which psilocybin fails to provide efficacy? Although some studies have reported sex-specific effects of psilocybin in rodents, the existing literature offers limited and inconsistent evidence regarding sex-based pharmacodynamic differences^29–31^. Accordingly, we employed a single-sex design to minimize potential confounding variables associated with sex-based biological differences and to ensure reproducibility in this initial in vivo investigation. Future studies are warranted to evaluate sex-specific therapeutic and mechanistic effects of psilocybin. Our in vitro studies suggest that psilocin-mediated cellular lifespan extension did not result in oncogenic transformation, as psilocin-treated cells did reach replicative senescence and exhaustion of proliferative potential. However, it could be argued that delayed exhaustion of proliferative potential and/or senescence could impact oncogenesis or cancer progression. Future research should rigorously assess the potential impacts of long-term psilocybin treatment in vivo on cancer incidence and/or progression. Few studies have evaluated the impacts of long-term prolonged dosing.

Beyond its neurological and psychological benefits, our findings suggest that psilocybin influences systemic aging processes, potentially explaining its long-lasting therapeutic effects across multiple disease indications. Although the impact of psilocybin on peripheral organs remains largely unexplored, these studies implicate untapped therapeutic potential for psilocybin’s systemic impacts. Psilocybin may represent a “disruptive” pharmacotherapy as a novel geroprotective agent to promote healthy aging and/or as a potential therapeutic intervention for age-related diseases.

## Methods

### Reagents

Psilocybin (catalog #14041) and psilocin (catalog # 11864) were obtained from Cayman Chemical (Ann Arbor, MI) under a DEA license. We purchased the following antibodies: GAPDH, RB, phosphor-RB, (Cell Signaling); Nox4, PCNA, p53, p21, and β-actin (Abcam); p16 (BD Biosciences); Secondary horseradish peroxidase (HRP)-conjugated anti-mouse and anti-rabbit antibodies (Bio-Rad). We purchased a Halt Protease and Phosphatase inhibitor cocktail (ThermoFisher Scientific). All other chemicals/reagents were purchased (Sigma) unless otherwise stated.

### Animal studies

Aged (19-month-old) female C57BL/6J mice were obtained from The Jackson Laboratory and acclimated in the institutional animal facility for one month prior to study initiation. Mice were then randomly allocated to control and treatment groups to ensure balanced group assignments, including comparable average body weights across groups. The rationale for dosing regimen utilized was based on a number of factors. First, we sought to model high-dose used in a clinical study for chronic pain, where patients were administered a psychedelic dose (25 mg) of psilocybin^24^. Using the standard allometric scaling method^32^, a human dose of 25 mg of psilocybin translates to a mouse dose of 5.14 mg/kg; this informed our starting point for dosing. However, mice exhibit a significantly faster metabolic profile for psilocybin compared to humans, leading to a shorter half-life and more rapid systemic clearance of psilocin; the elimination half-life of psilocin is ~0.9 h in mice^33^ vs 1.8–3 h in humans^34^. Due to this rapid clearance in mice, a higher dose of 15 mg/kg was selected to ensure sufficient systemic exposure comparable to those observed in human clinical trials. It is also important to note that toxicology studies indicate that psilocybin is well tolerated in mice up to doses of 180–250 mg/kg^35^, which is well above the dose utilized in this study. Mice received a low-dose (5 mg/kg) initially for the first treatment to acclimate mice for long-term treatment, followed by monthly high-dose of psilocybin (15 mg/kg in sterile saline) or vehicle (sterile saline) via oral gavage (on conscious mice) once/month (10 treatments total) (Fig. 2A); all treatments were administered monthly between ~9:00am and 12pm. Mice received treatments from a stock concentration of drug (4 mg/ml in sterile saline); mice were weighed on each treatment day and received a gavage volume ranging from 100 to 200 μl (total volume), depending on their weight at the time of treatment. All mice were sacrificed once any group reached 50% mortality, signaling the end of the study per IACUC protocol. Mice were euthanized by CO_2_ inhalation followed by a secondary method to ensure death, in accordance with the American veterinary medical association (AVMA) guidelines for the euthanasia of animals and approved institutional IACUC protocols. Mice were monitored for body weight and signs of morbidity throughout the duration of the experiment. Mice were provided standard chow ad libitum and maintained under a 12:12-h light/dark cycle. Researchers were not blinded to group allocation during the experimental procedures or data analysis due to logistical constraints, including regulatory and safety protocols associated with handling psilocybin, a Schedule I controlled substance, as well as the exploratory nature of the study. All experiments and procedures involving animals were conducted in accordance with Institutional Animal Care and Use (IACUC) Committee guidelines at Emory University (PROTO202000138).

### Telomere length assay

Genomic DNA was isolated from cells using the QIAwave DNA Blood & Tissue kit (QIAGEN). Quantitative RT-PCR was performed with genomic DNA using primers designed against human telomere sequence or a single copy reference gene (Human kit, ScienCell, Catalog #8918). The single copy reference (SCR) primer set recognizes and amplifies a 100 bp-long region on human chromosome 17, which serves as reference for data normalization; Amplification curves were compared to the reference control with a known telomere length.

### Cell culture

Human fetal lung fibroblasts (IMR-90) were purchased (Coriell Cell Repositories) and adult skin fibroblasts were purchased (ATCC). IMR-90 cells were cultured in DMEM supplemented with 10% fetal bovine serum (FBS), 100 U/ml penicillin, 100 μg/ml streptomycin. Skin fibroblasts were cultured in fibroblast basal media supplemented with 2% FBS, L-glutamine (7.5 mM), FGF (5 ng/ml), insulin (5 μg/ml), hydrocortisone (1 μg/ml), and ascorbic acid (50 μg/ml). All cells were purchased at passages 7–8 and expanded for 2–3 passages prior to initiating treatment protocols. Psilocin was dissolved in DMSO (50 mM stock) for in vitro studies.

### Population doubling

Cells were plated at a fixed density (1 × 10^6^) and cultured in a T75 flask. Cells were counted using a TC20 cell counter (Bio-rad) and passaged every 3–4 days and re-plated (1 × 10^6^) throughout the duration of the experiment (until cells reached replicative senescence). The number of population doublings (PD) was calculated. We quantified the differences of psilocin vs. vehicle treatment using the Area Under the Growth Curve (AUC), as an indicator of treatment effect on cell doubling^36,37^.

### Senescence assays

We used β-galactosidase substrate for the quantitative assessment of cellular senescence (ThermoFisher Scientific), according to the manufacturer’s instructions. We also used a senescence detection kit designed to histochemically detect β-gal activity in cultured cells (Abcam).

### Western immunoblotting

We prepared cell lysates in RIPA buffer with Halt protease and phosphatase inhibitor, subjected them to SDS-PAGE under reducing conditions, and performed western immunoblotting as previously described. Lysates were quantitated using a Micro BCA Protein assay kit (Pierce) according to instructions. We used ECL western blotting substrate (Azure biosystems) and Azure C400 Imaging Systems (Azure biosystems) to detect specific immunoreactive signals.

### Reactive oxygen species (ROS) detection

Hydrogen peroxide (H_2_O_2_) levels in cells was evaluated by Amplex Red assay kit (ThermoFisher Scientific). The fluorescence intensity was measured at 550 nm for excitation and emission in the range of 590 nm using the Synergy H1 Plate Reader (BioTek).

### Statistical analysis

Graphs were generated and statistical analyses were performed with GraphPad Prism Software *Version 10.4.1 (532) Boston, MA, USA*. For all cell culture experiments (Fig. 1), population doubling levels were calculated based on direct cell counts performed in four technical replicates at each passage. These replicate values were used to compute the mean ± standard deviation (SD) for each time point. Data from other assays, including β-galactosidase activity, ROS production, and telomere length, were analyzed using 3–5 technical replicates per group, as specified in the figure legends. Statistical comparisons were performed using two-tailed unpaired *t*-tests with unequal variance, or two-way ANOVA where appropriate. For the animal survival study (Fig. 2), survival curves were analyzed using the log-rank Mantel–Cox test. To ensure uniform survival endpoint analysis, all mice were euthanized at 10 months post-initial treatment, when the first group reached median survival. A *p*-value less than 0.05 was considered statistically significant. Complete details of statistical tests, number of replicates (*n*), and significance thresholds are provided in figure legend.

## Supplementary information

Supplementary Figure

## Data Availability

All datasets generated and/or analyzed during the current study are publicly available within this manuscript or the supplemental material.
