# Supplementary material for: Psilocybin treatment extends cellular lifespan and improves survival of aged mice
Source: NPJ Aging. 2025 Jul 8;11(1):55. doi: 10.1038/s41514-025-00244-x (PMC12238350; doi:10.1038/s41514-025-00244-x)
Supplement: Supplementary file 1 — Supplementary Figure [file 41514_2025_244_MOESM1_ESM.pdf]

Supplemental Figures

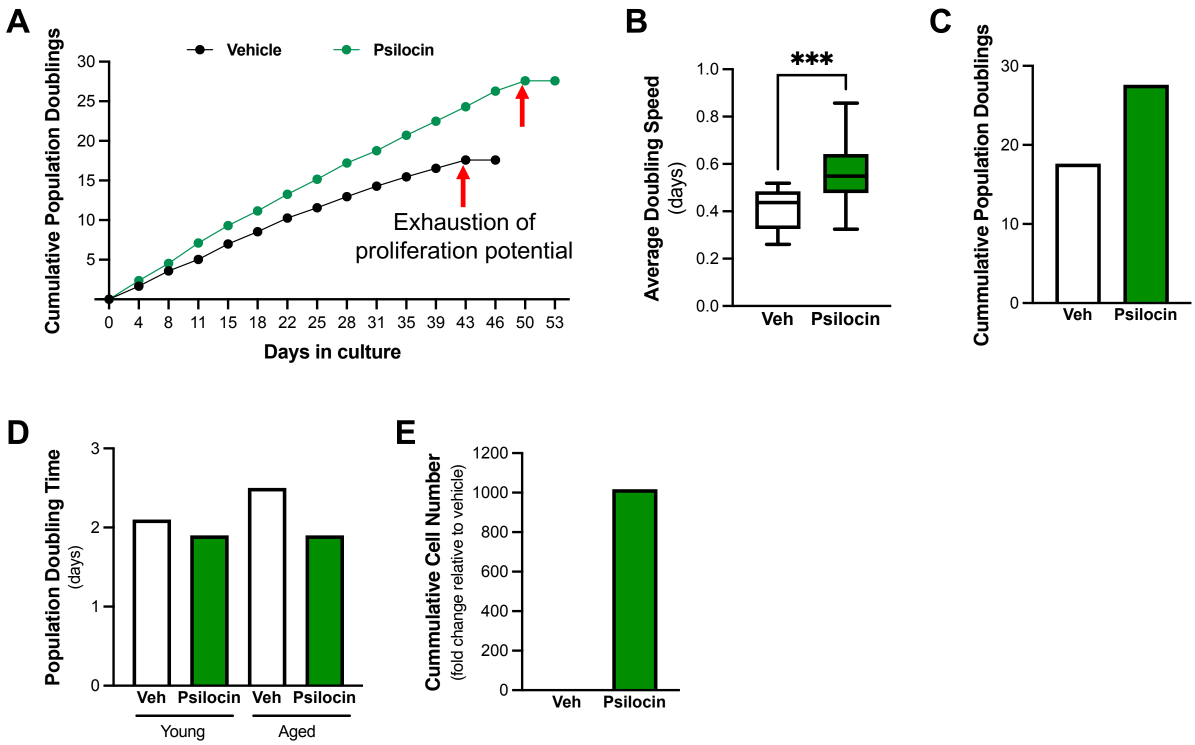

F

Cumulative Population Doublings			Lifespan
**Vehicle**	**Psilocin (100µM)**	**Extension**	**% of Extension**
**17.6**	**27.6**	**10**	**56.8**

Supplemental Figure 1. High-dose psilocin treatment extends cellular lifespan.

Human lung fibroblasts were treated continuously with vehicle (DMSO 0.2%) or 100µM psilocin until they reached replicative senescence. (**A**) Cumulative population doubling curves; arrows indicate time point when cells reach replicative senescence. (**B**) The speed of population doubling time was quantified for each timepoint interval. Significance of differences was evaluated by doubling speed for each treatment group using a two-sided unequal variance t-test; ***$p < 0.001$. (**C**) Cumulative population doublings at onset of senescence. (**D**) Population doubling time

comparing young cells (0-4 days) vs. aged cells (39-43 days) post-treatment. (**E**) Cumulative cell number over cellular lifespan. (**F**) Table showing cellular lifespan population doublings and lifespan extension. Extension refers to the number of additional population doublings of psilocin-treated cells vs. vehicle, which is also shown as a percent increase relative to vehicle-treated cells.

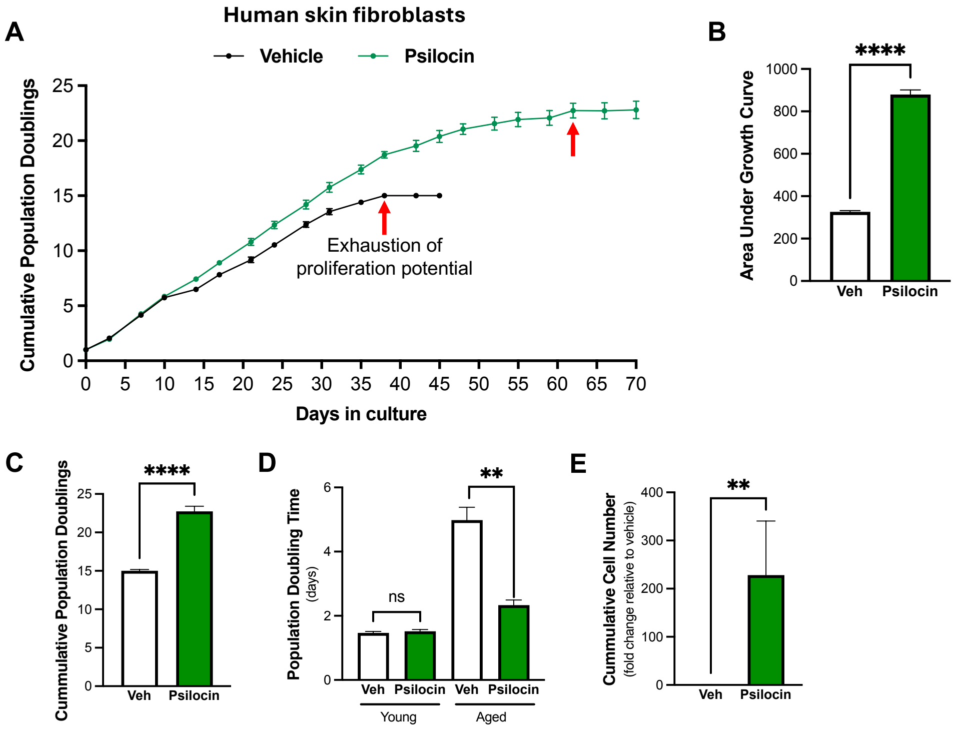

F

Cumulative Population Doublings			Lifespan
Vehicle	Psilocin (100µM)	Extension	% of Extension
15.0 ± 0.2	22.7 ± 0.7	7.7 ± 0.7	51.5 ± 4.4

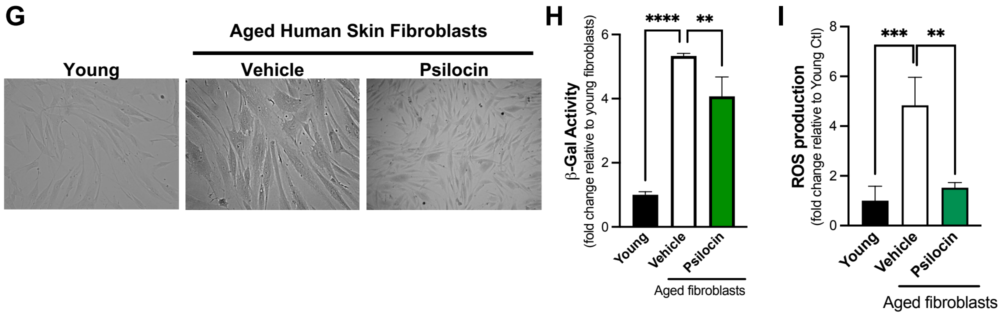

<u>Supplemental Figure 2</u>. Psilocin treatment extends cellular lifespan in adult skin fibroblasts.

Adult skin fibroblasts were treated continuously with vehicle (DMSO 0.2%) or 100µM psilocin until they reached replicative senescence. (**A**) Cumulative population doubling curves; arrows indicate time point when cells reach replicative senescence. (**B**) The area under the curve (AUC) was

calculated using the sum of trapeze areas for each time point intervals. Significance of differences in AUC between treatment groups were assessed using a two-sided unequal variance t-test, with significance achieved at $p<0.05$. (**C**) Cumulative population doublings at onset of senescence. (**D**) Population doubling time comparing young cells (0-3 days) vs. aged cells (35-38 days) post-treatment. Groups were compared using 2-way ANOVA; **$p < 0.01$, ns = not significant. (**E**) Cumulative cell number over cellular lifespan. (**F**) Table showing cellular lifespan population doublings and lifespan extension. (**G**) Cellular morphology is shown. (**H**) Senescence was assessed by quantitative assessment of β-gal activity. (**I**) ROS production was evaluated by Amplex Red assay. All values represent means ± SEM (n=3-5 technical replicates). For B-C, E, H-I, groups were assessed using a two-sided unequal variance t-test; **$p < 0.01$, ***$p < 0.001$, ****$p < 0.0001$, ns = not significant.

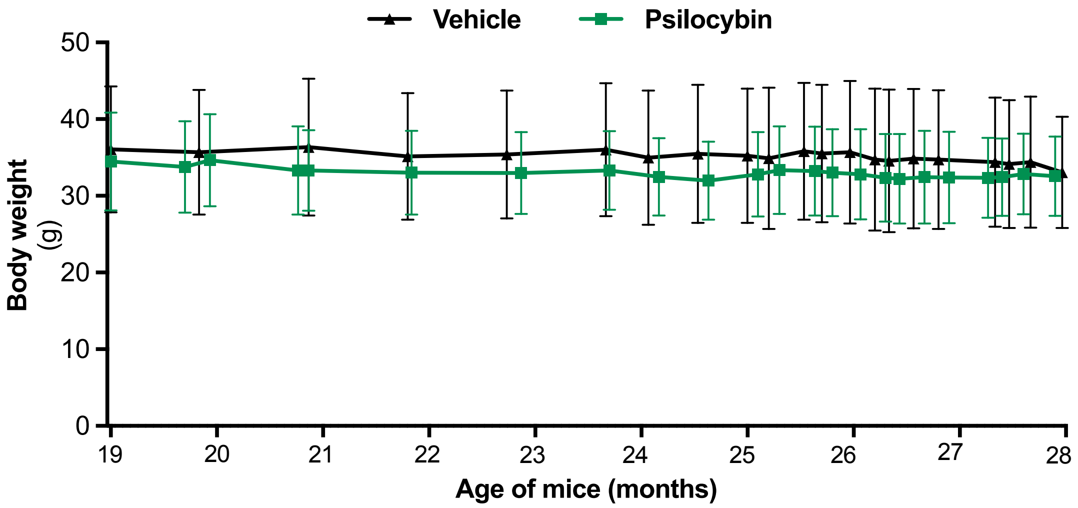

Supplemental Figure 3. Body weight of vehicle- and psilocybin-treated mice was recorded throughout the duration of the treatment protocol. Data shown represent all surviving mice at the conclusion of the study. Data are presented as mean ± SEM.

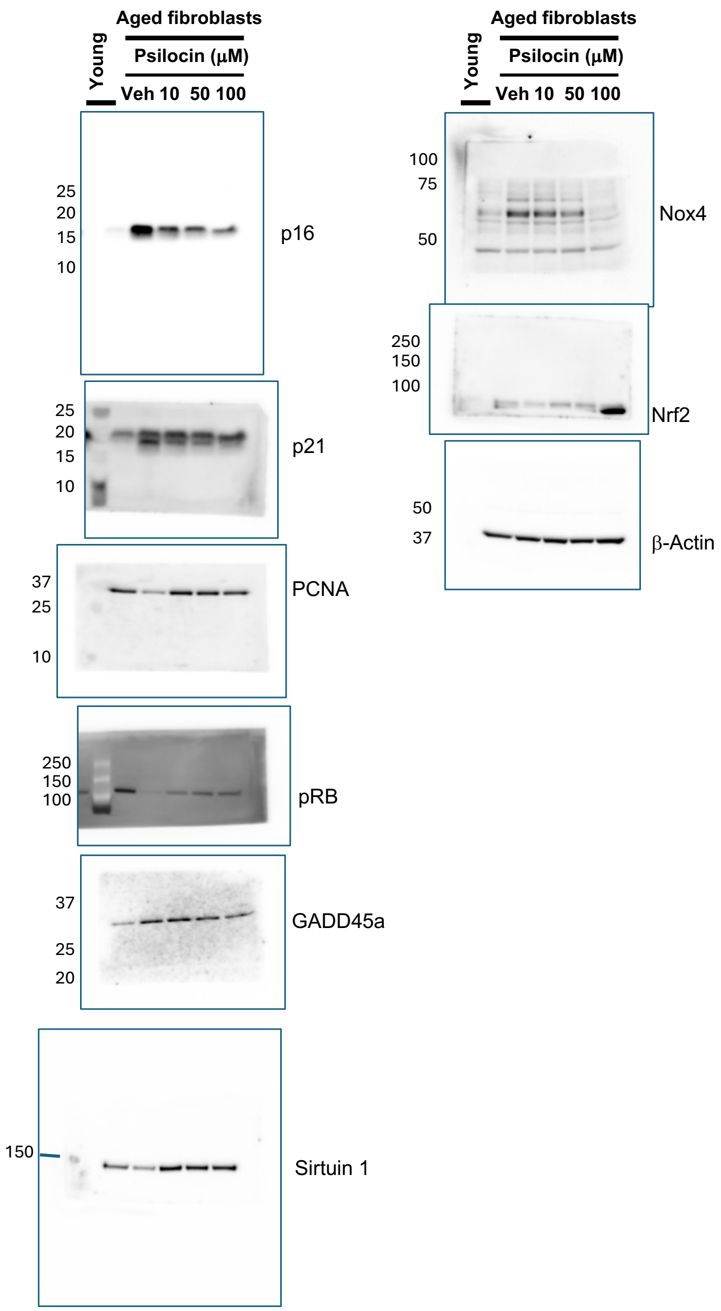

<u>Supplemental Figure 4</u>. Uncut western blot images. Uncut western blot images from Figure 1 are shown.
